# Supplementary material for: Charge decay in the spatial afterglow of plasmas and its impact on diffusion regimes
Source: Nat Commun. 2023 Nov 2;14:6776. doi: 10.1038/s41467-023-42442-9 (PMC10622414; doi:10.1038/s41467-023-42442-9)
Supplement: Supplementary file 1 — Supplementary Information [file 41467_2023_42442_MOESM1_ESM.pdf]

Supplementary Information

Charge decay in the spatial afterglow of plasmas and its impact on diffusion regimes

Nabiel H. Abuyazid[1], Necip B. Üner[2,3], Sean M. Peyres[2], and R. Mohan Sankaran[2]

[1]*Department of Chemical and Biomolecular Engineering, University of Illinois, Urbana, USA.*

[2]*Department of Nuclear, Plasma and Radiological Engineering, University of Illinois, Urbana, USA.*

[3]*Chemical Engineering Department, Middle East Technical University, Ankara, Turkey*

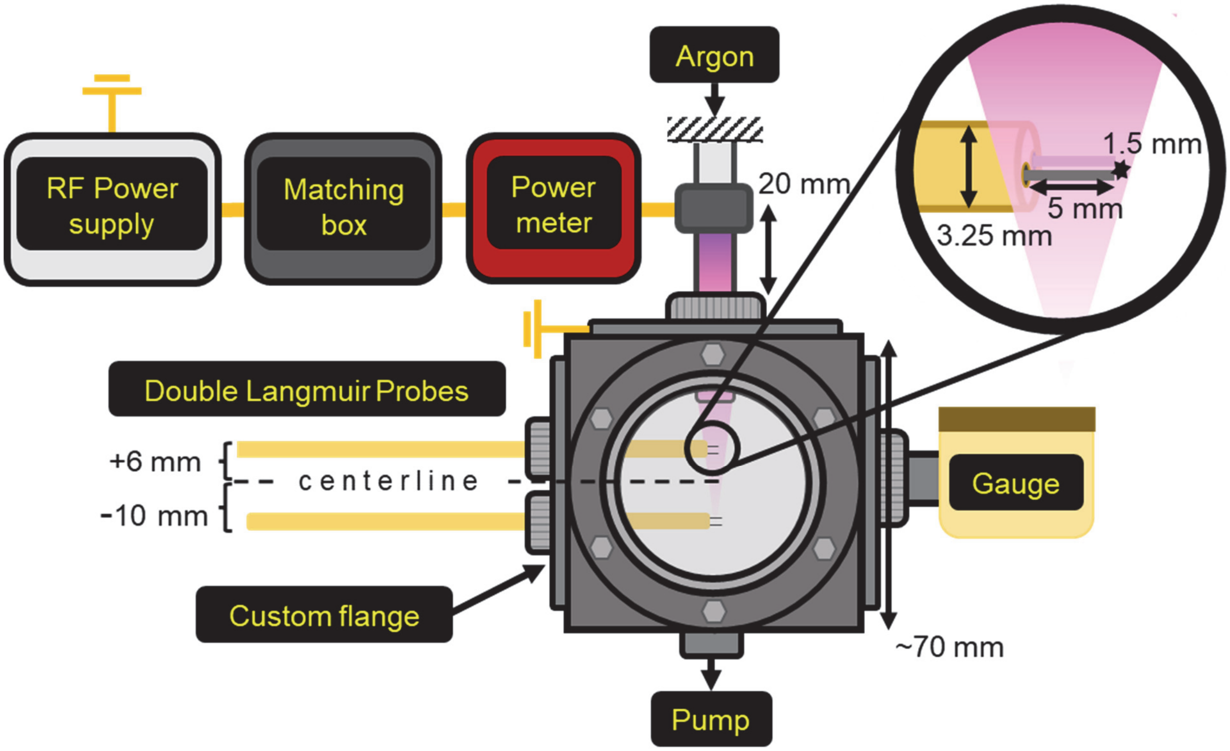

Supplementary Figure 1. Schematic of experimental setup for generation and characterization of spatial afterglow. The setup consists of a radio frequency (RF)-powered bulk plasma reactor and a spatial afterglow chamber. A double Langmuir probe (depicted in detail in the inset) provides spatial measurements of the charged species density in the spatial afterglow.

Supplementary Note 1. Equivalent circuit model of bulk plasma to obtain electron density. Electrical measurements of the driving circuit and a fluid plasma model were used to obtain the electron density of the bulk plasma. Briefly, the plasma resistance, R_p, is calculated from the conduction current and the discharge voltage. The conduction current was calculated by subtracting the displacement current from the total current measured from the bulk plasma. Measuring the current while the plasma was on provided the total current in the system, and measuring the current while the plasma was off provided a rough estimate of the displacement current. An equivalent circuit to represent the power transmission system (denoted with subscript x), the plasma reactor (denoted with subscript p), and the stray components (denoted with subscript s) was constructed, as depicted in Supplementary Fig. 2. Stray contributions here refer to the resistance and capacitance of the quartz tube. When the plasma is off, there is only power transmission and stray components, and when the plasma is on, the plasma components are in parallel with the stray components.

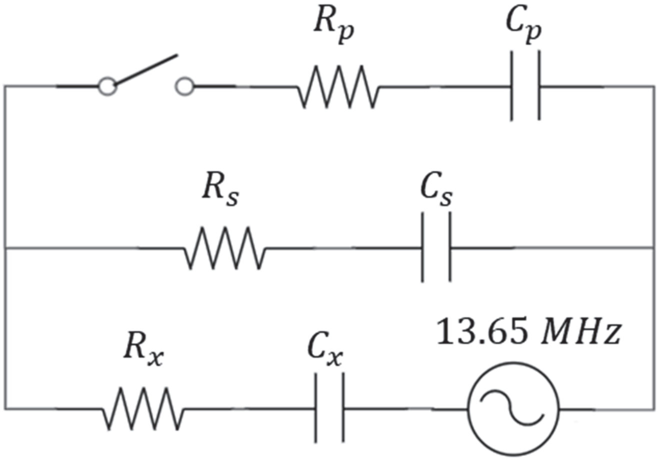

Supplementary Figure 2. Equivalent circuit for an axisymmetric plasma reactor. Schematic of equivalent circuit constructed to obtain the plasma density from electrical measurements consisting of the power transmission system and the plasma reactor, the latter of which includes plasma and stray components (p = plasma, s = stray, and x = transmission).

The analysis of the circuit begins with the expression for the total impedance when the plasma is switched on. The circuit impedance is composed of two parallel contributions, the plasma impedance, Z_p, and the stray impedance, Z_s. The total system impedance, Z_{on}, can be expressed as:

$$Z_{\mathrm{on}} = \frac{V_{\mathrm{A}}}{I_{\mathrm{A}}} = \left(\frac{1}{Z_{\mathrm{p}}} + \frac{1}{Z_{\mathrm{s}}}\right)^{-1} \tag{S1}$$

where V_{A} is the measured 'plasma on' voltage and I_{A} is the measured 'plasma on' complex current. From the plasma off equivalent circuit, we can obtain an expression for the stray impedance,

$$Z_{\mathrm{s}} = \frac{V_{\mathrm{B}}}{I_{\mathrm{B}}} \tag{S2}$$

where V_{B} is the measured 'plasma off' voltage, and I_{B} is the measured 'plasma off' complex current. Combining Equations S1 and S2, we can obtain an expression for the plasma impedance Z_{p},

$$Z_{\mathrm{p}} = \left(\frac{I_{\mathrm{A}}}{V_{\mathrm{A}}} - \frac{I_{\mathrm{B}}}{V_{\mathrm{B}}}\right)^{-1} = \frac{V_{\mathrm{A}}V_{\mathrm{B}}}{V_{\mathrm{B}}I_{\mathrm{A}} - V_{\mathrm{A}}I_{\mathrm{B}}}. \tag{S3}$$

The measured current has a real and an imaginary component. The products of current and voltage above can be represented as real and imaginary parts for simplicity: $V_{\mathrm{B}}I_{\mathrm{A}} = a + bj$ and $V_{\mathrm{A}}I_{\mathrm{B}} = c + dj$, where j is the imaginary unit. Substitution of the complex notation into Equation S3 results in the final expression for Z_{p},

$$Z_{\mathrm{p}} = \frac{V_{\mathrm{A}}V_{\mathrm{B}}(a - c)}{(a - c)^2 + (b - d)^2} - \frac{V_{\mathrm{A}}V_{\mathrm{B}}(b - d)}{(a - c)^2 + (b - d)^2} j \tag{S4}$$

where only real part of the plasma impedance is then used for the fluid plasma model result (Equation 5 in the main text),

$$R_{\mathrm{p}} = \frac{V_{\mathrm{A}}V_{\mathrm{B}}(a - c)}{(a - c)^2 + (b - d)^2}. \tag{S5}$$

The RF power probe (Octiv Poly, Impedans Ltd.) provided measurements of the voltage and complex current, which are then used to evaluate the plasma resistance, R_{p}, using Equation S5.

Supplementary Note 2. Spatially-resolved measurements of spatial afterglow using a double Langmuir probe. In this study, double Langmuir probes (DLPs) were used to characterize the spatial afterglow. DLPs are floating probes and do not introduce a resistive path to ground for charged species, and thus minimally disrupt the plasma, or in this case, the spatial afterglow[1–4]. In DLP measurements, an external voltage is applied between two probe tips, which are connected to a floating circuit. As a result, both probes are charged negatively with respect to the plasma potential, but one more negative than the other. At zero applied voltage, both probes collect the electrons that can overcome the floating potential of the tips. As the voltage difference increases, the more negative probe collects less electrons, but more ions, whose current is limited by the ion concentration in the probe sheath. At very high applied voltages (positive or negative), the total current in the DLP circuit is fully determined by the ion current. By applying a voltage sweep from negative to positive few tens of volts, a symmetric current-voltage (I-V) curve is measured with ion saturation regions at high applied voltages and an electron retarding region at small voltages. Analysis of these regions provides estimates for the ion density and electron temperature, respectively.

DLP can directly measure the electric field strength in a plasma. As a result of the local electric field, current flows through the unbiased probes, and the symmetry center of the I-V curve is shifted away from the origin. Thus, the degree of shift in the x-axis of the curve gives the difference in plasma potential, and knowing the distance between the probes, the electric field strength is obtained.

Theory shows that the characteristic sigmoidal shape of DLP traces observed in ideal, low-pressure systems is maintained at high pressures up to atmospheric, making it possible to apply drift-diffusion equations to describe ion and electron transport to the probe tips[5]. The theory has been previously validated by several experiments on plasmas and flames[6,7]. To further support, we calculated the ratio of the electron mean free path to the Debye length, which was found to be $>10^2$ at all pressures studied, confirming that the electrons can be assumed to be collisionless.

It is important to note that the spatial resolution of DLP measurements depends on the dimensions of the conducting tips. Here, probes were constructed from a 3.25 mm (1/8" nominal) O.D. alumina

tube with a double-bore large enough to accommodate a 0.5 mm stainless steel wire as probe tips. Alumina-based ceramic adhesive (16026, PELCO) was used to fill in the bores on the plasma-facing end and limit the amount of conductive area exposed to the plasma. The probe tips were cut to 5 mm in length and the separation between probe tips varied between 1.15 and 1.5 mm. Epoxy resin (Torr Seal, Agilent) was applied to the other side to ensure that the probe was vacuum tight.

Supplementary Figure 3 shows representative DLP I-V traces. Two key features of the I-V trace are marked: the ion saturation region and the electron retardation region. The ion saturation region is used to determine the ion saturation current, $I_{\mathrm{i,sat}}$, by extrapolating the ion current, I_+, in the ion saturation region towards the y-axis and finding the intercept. This procedure accounts for the sheath expansion caused by increasing probe potential and is applied to both ion saturation regions. The obtained intercept values are averaged and referred to as the ion saturation current. An ion collection model is needed to interpret the ion saturation current to obtain ion density in the local vicinity of the probe. We followed the analytical expression for ion collection by a cylindrical probe reported by Iza and Lee.[8] Their model combines the free fall of ions in the sheath with ion-neutral collisions and ion scattering. The ion density, n_{i}, is then related to ion saturation current, $I_{\mathrm{i,sat}}$, by:

$$n_{\mathrm{i}} = \frac{I_{\mathrm{i,sat}}}{Sq_{\mathrm{e}}} \frac{\left(1 + \frac{5\lambda_{\mathrm{D}}}{\lambda_{\mathrm{i,mfp}}}\right)}{0.57u_{\mathrm{Bohm}}} \tag{S6}$$

where S is the surface area of the probe tip, q_{e} is the elementary charge, $u_{\mathrm{Bohm}} = (k_{\mathrm{B}}T_{\mathrm{e}}/M_{\mathrm{i}})^{1/2}$ is the Bohm velocity, λ_{D} is the Debye length, and $\lambda_{\mathrm{i,mfp}} = k_{\mathrm{B}}T_{\mathrm{i}}/\sqrt{2}P\sigma$ is ion mean free path where P is the pressure and σ is the ion collision cross-section (~10^{-17} m^2 for argon)[9]. Collisionality in the sheath leads to ion approach velocities smaller than the Bohm velocity by a factor of ($1 + 5\lambda_{\mathrm{D}}/\lambda_{\mathrm{i,mfp}}$). Assuming quasi-neutrality, the measured ion density is equal to the electron density.

The electron retardation region corresponds to the steep portion of the I-V trace and is analyzed to obtain the electron temperature. Collisionality between electrons and neutrals can complicate the analysis of this region by affecting the energy distribution of electrons. However, since the ratio of the electron mean free path to the Debye length is much greater than one (~3000 at 10 Torr and ~800 at 300 Torr), the electrons are effectively collisionless near the probe tips for the conditions

considered in this investigation. Thus, the electron temperature can be extracted from the slope of this region where a small fraction of the electrons can reach the probe tips:[10]

$$T_e^{-1} = (I_{\text{i,sat}})^{-1}\left[\left(\frac{dI_{+1}}{dV_{\text{p1}}}\right)_0 - 2\left(\frac{dI}{dV}\right)_0\right] \quad \text{(S7)}$$

where T_e is the electron temperature in eV, I_{+1} is the ion current, and V_{p1} is probe 1 voltage. The first term in the square bracket is the slope of the ion saturation current extrapolated to $V = 0$, and the second term is the slope of the I-V curve about the symmetry point.

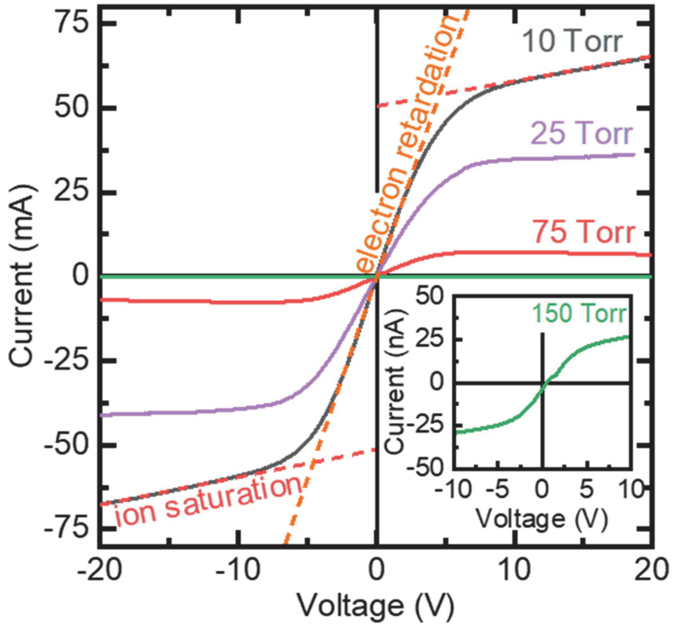

Supplementary Figure 3. Double Langmuir probe measurements of spatial afterglow. Representative double Langmuir probe (DLP) traces of a spatial afterglow collected at 10, 25, 75 and 150 Torr. The inset shows a magnified view of the trace at 150 Torr. The dotted lines highlight the ion saturation region that occurs at relatively large applied potentials and the electron retarding region that occurs at small applied potentials. Traces were corrected for both axial and lateral shifts, which can occur due to slight differences in plasma potential nearby each tip and tip dimensions. Measurements were taken at the closest axial position to the bulk plasma (i.e., 0 cm relative distance).

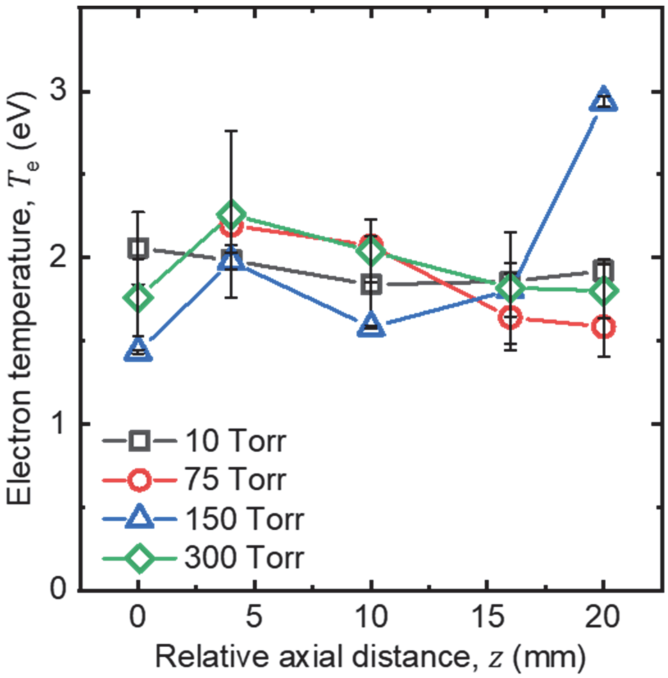

Supplementary Figure 4. Electron temperature in spatial afterglow. Electron temperatures as a function of axial distance in the spatial afterglow obtained by double Langmuir probe measurements at 10, 75, 150 and 300 Torr. Data points represent an average of five measurements and error bars correspond to one standard deviation.

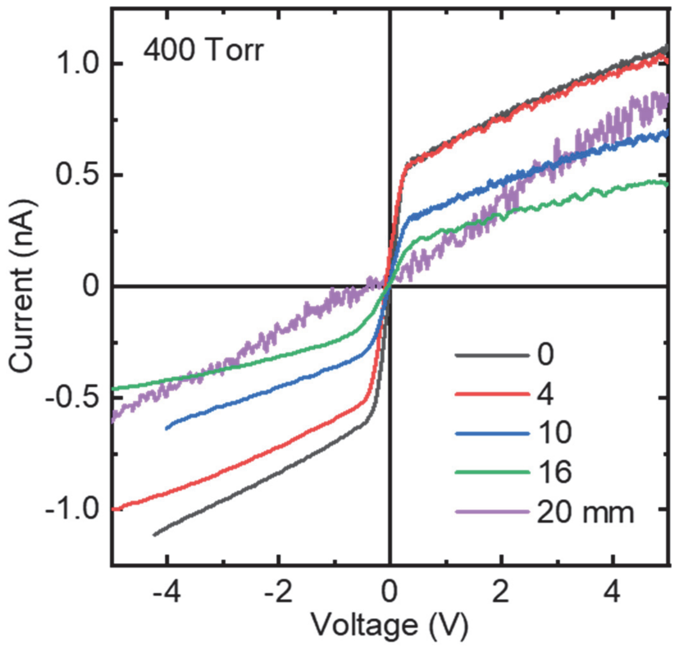

Supplementary Figure 5. Double Langmuir probe measurements of spatial afterglow at 400 Torr. Double Langmuir probe traces of spatial afterglow as a function of axial distance at a constant pressure of 400 Torr. At this pressure, the characteristic sigmoidal shape of the *I*-*V* trace eventually disappears far away from the bulk plasma. The forward RF power in the bulk plasma was 20 W and the gas flow rate was 1000 sccm. Savitzky-Golay smoothing was applied to the data (20 points per window, 3rd order polynomial). Traces were corrected for the origin shift as explained in Supplementary Figure 3.

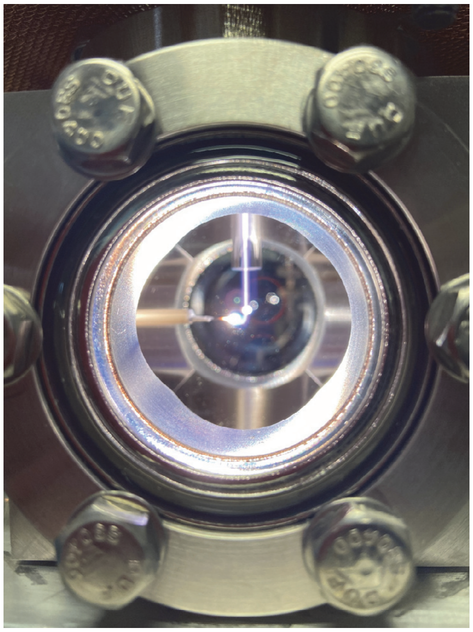

Supplementary Figure 6. Single Langmuir probe measurements of spatial afterglow. Photo of single Langmuir probe in spatial afterglow at 150 Torr showing coupling to the bulk plasma that prevents formation of the afterglow and leads to probe damage.

Supplementary Note 3. Power dissipation model. To estimate the length of the bulk plasma, i.e., the plasma-afterglow boundary at different pressures, we related the power input to various dissipative processes. In general, the electrical power absorbed by a plasma is dissipated as heat via ionization, leading to an increase in the enthalpy of the gas flow and heat loss to the surroundings. Specifically, the heat generation mechanisms include heat generation by electron-neutral elastic collisions and three-body recombination in the volume, and recombination on the surface of the walls. Assuming that the power dissipated by these mechanisms is additive and at steady state, we can write out the power balance as:

$$P_{\mathrm{w}}^{\mathrm{input}} = P_{\mathrm{w}}^{\mathrm{el}} + P_{\mathrm{w}}^{\mathrm{r}} + P_{\mathrm{w}}^{\mathrm{s}} \quad \text{(S8)}$$

where $P_{\mathrm{w}}^{\mathrm{input}}$ is the input power and $P_{\mathrm{w}}^{\mathrm{el}}$, $P_{\mathrm{w}}^{\mathrm{r}}$ and $P_{\mathrm{w}}^{\mathrm{s}}$ represent the power dissipation due to elastic collisions, three-body recombination, and surface recombination, respectively.

The power dissipation density resulting from elastic collisions between electrons and neutral gas atoms in the entire volume of the bulk plasma can be expressed as[11]:

$$P_{\mathrm{w}}^{\mathrm{el}} = \left(\frac{2m_{\mathrm{e}}}{M}\right) \varepsilon_{\mathrm{mean}} n_{\mathrm{p}} \nu_{\mathrm{el}} \quad \text{(S9)}$$

Where R is the radius of the cylindrical reactor, L is the length of the bulk plasma, m_{e} is the mass of electrons, M is the mass of neutral gas atoms, $\varepsilon_{\mathrm{mean}} = 3k_{\mathrm{B}}T_{\mathrm{e}}/2$ is the average energy of the electrons, n_{g} is the neutral gas density, n_{p} is the bulk plasma density, and ν_{el} is the electron-neutral elastic collision frequency, which is expressed as:

$$\nu_{\mathrm{el}} = n_{\mathrm{g}} \sqrt{\frac{\pi \alpha_{\mathrm{R}} a_0^3 q_{\mathrm{e}}^2}{\epsilon_0 m_{\mathrm{e}}}} \quad \text{(S10)}$$

where α_R is the relative polarizability of the neutral gas, a_0 is the Bohr radius, q_{e} is the elementary charge, and ϵ_0 is the vacuum permittivity.

Because we are interested in the effect of pressure, we can use the empirical description of $n_{\mathrm{p}}(p) = \beta p$ shown in Fig. 2a in the main text and the ideal gas law to explicitly write the dependence of the power dissipation due to elastic collisions as:

$$P_{\mathrm{w}}^{\mathrm{el}} = 3\left(\frac{m_{\mathrm{e}}}{M}\right)\sqrt{\frac{\pi\alpha_{\mathrm{R}}a_0^3q_{\mathrm{e}}^2}{\epsilon_0 m_{\mathrm{e}}}}\frac{\beta T_{\mathrm{e}}}{T_{\mathrm{g}}}p^2 \quad \text{(S11)}$$

Next, we can similarly express the power dissipation density due to three-body recombination in the plasma volume as:

$$P_{\mathrm{w}}^{\mathrm{r}} = \Delta H_{\mathrm{rec}}k_{\mathrm{r}}n_{\mathrm{g}}n_{\mathrm{p}}^2 \quad \text{(S12)}$$

where ΔH_{rec} is the heat of recombination for argon and k_r is the three-body recombination coefficient. Again, assuming ideal gas and $n_p(p) = \beta p$, we can express it as an explicit function of pressure:

$$P_{\mathrm{w}}^{\mathrm{r}} = \Delta H_{\mathrm{rec}}k_{\mathrm{r}}\beta^2 p^3 \quad \text{(S13)}$$

We note that the contribution of volumetric three-body recombination to power dissipation was found to be much smaller than elastic collisions and was therefore neglected in our calculations. We show this more explicitly when we present the heat model later (see Supplementary Figs. 9-11).

Finally, the power dissipated due to surface recombination is limited by the rate of ambipolar diffusion to the walls:

$$P_{\mathrm{w}}^{\mathrm{s}} = \Delta H_{\mathrm{rec}}\frac{2D_{\mathrm{a}}}{R}n_{\mathrm{p}} \quad \text{(S14)}$$

where D_a is the ambipolar diffusion coefficient. Using $D_a = D_a^0 p^0/p$ and $n_p(p) = \beta p$, we find that the power dissipation surface density due to surface recombination is independent of pressure:

$$P_{\mathrm{w}}^{\mathrm{s}} = \frac{2\Delta H_{\mathrm{rec}}D_{\mathrm{a}}^0 p^0 \beta}{R} \quad \text{(S15)}$$

where D_a^0 is the ambipolar diffusion coefficient at reference pressure p^0.

Using Equations S11, S13 and S15, we can solve for the length of the plasma, L, from Equation S8. However, these equations are general, and in the actual experiment, the length of the plasma is physically limited in two ways by: 1) the distance between the powered and ground electrodes, L_0, which is equal to 20 mm; and 2) the length of the quartz tube from the powered electrode to where it ends in the vacuum chamber, L_{max} which is equal to 63.5 mm. These physical dimensions represent the minimum length the plasma can occupy (L_0), filling the gap between the electrodes,

and the maximum length the plasma can occupy (L_{max}), where the reactor for the bulk plasma ends. Between these values, power dissipation governs the expansion of the plasma volume, and we can estimate the actual length, L, as:

$$L = \left(\frac{1}{L_{\mathrm{p}}} + \frac{1}{L_{\mathrm{max}}}\right)^{-1} \geq L_0 \tag{S16}$$

where,

$$\frac{1}{L_{\mathrm{p}}} = \frac{\left[\pi R^2 \left(P_{\mathrm{w}}^{\mathrm{el}} + P_{\mathrm{w}}^{\mathrm{r}}\right) + 2\pi R \left(P_{\mathrm{w}}^{\mathrm{s}}\right)\right]}{P_{\mathrm{w}}^{\mathrm{input}}} \cong \frac{\pi R^2 P_{\mathrm{w}}^{\mathrm{el}} + 2\pi R P_{\mathrm{w}}^{\mathrm{s}}}{P_{\mathrm{w}}^{\mathrm{input}}} \tag{S17}$$

Finally, we can define the length of the plasma beyond the ground electrode, γ_0, which is important for comparison between the plasma decay model and DLP measurements:

$$\gamma_0 = \left\{\frac{1}{P_{\mathrm{w}}^{\mathrm{input}}}\left[\pi R^2 \left(\frac{2m_{\mathrm{e}}}{M}\right)\sqrt{\frac{\pi \alpha_{\mathrm{R}} a_0^3 q_{\mathrm{e}}^2}{\epsilon_0 m_{\mathrm{e}}}}\frac{\beta T_{\mathrm{e}}}{T_{\mathrm{g}}} p^2 + 4\pi \Delta H_{\mathrm{rec}} p^0 D_{\mathrm{a}}^0 \beta\right] + \frac{1}{L_{\mathrm{max}}}\right\}^{-1} - L_0 \tag{S18}$$

Supplementary Note 4. Computational fluid dynamics (CFD) simulations. CFD was used to assess the expansion of the jet in the diagnostics chamber. Since Mach numbers were approximately around 0.3 for the lowest pressure investigated (10 Torr) and lower than 0.3 for higher pressures, compressible Navier-Stokes equations and the equation of continuity were used at room temperature:

$$\rho(\mathbf{u} \cdot \boldsymbol{\nabla})\mathbf{u} = \boldsymbol{\nabla} \cdot (-p\mathbf{I} + \mathbf{K}) \tag{S19}$$

$$\boldsymbol{\nabla} \cdot (\rho\mathbf{u}) = 0 \tag{S20}$$

where ρ is density, $\mathbf{u}$ is the velocity vector, p is pressure, $\mathbf{I}$ is the identity matrix, and $\mathbf{K}$ is the shear stress tensor. These equations were solved by using COMSOL Multiphysics v5.6 in an axisymmetric 2D domain. The flow geometry is illustrated in Supplementary Fig. 7a and a sample flow map at 50 Torr is shown in Supplementary Fig. 7b. The axial flow velocity at the centerline is shown in Supplementary Fig. 7c as a function of axial distance and pressure. The flow slightly decelerates due to expansion into the larger downstream of the tubular region, but as further seen in Supplementary Fig. 7d, the expansion is slight at all pressures, hence the average velocity and jet radius are approximately constant. Note that the radial width of the chamber is 2.5 cm, much larger than the radius over which the flow expands.

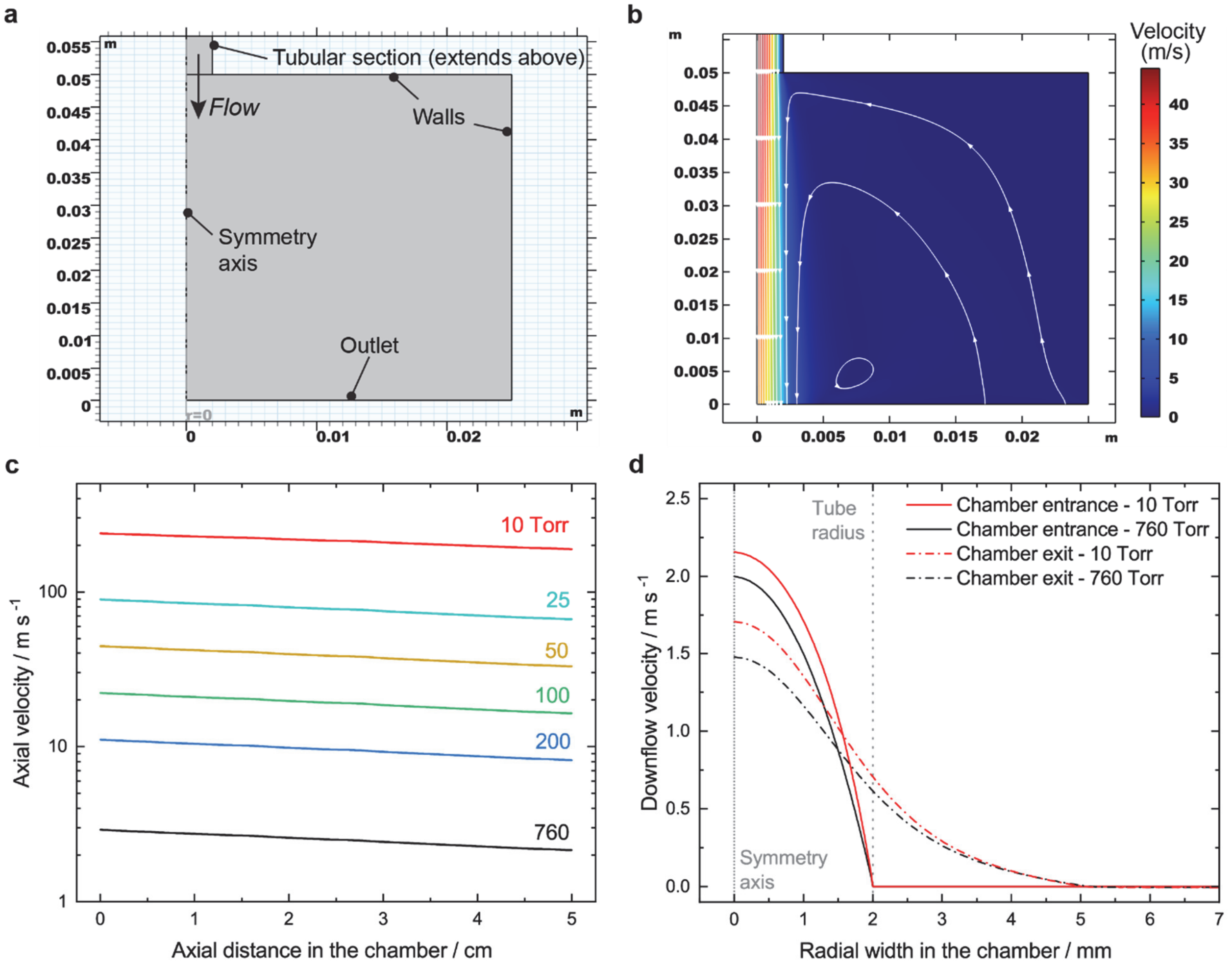

Supplementary Figure 7. Computational fluid dynamics simulations of spatial afterglow chamber. (a) Schematic diagram of simulation domain and boundaries. **(b)** Velocity field at 50 Torr showing there is negligible expansion beyond the radius of the tube. **(c)** Axial velocities at the centerline as a function of axial distance showing that the change is small (between 20%−50%) at all pressures studied. **(d)** Radial velocity distributions at the entrance and exit of the chamber (5 cm distance apart) at 10 and 760 Torr pressure.

Supplementary Note 5. Spatial afterglow decay model. Here, we provide additional details, including assumptions and derivation, of the advection-diffusion-recombination model used to calculate the decaying charge density in the spatial afterglow.

At the pressure range of interest (10-400 Torr), the flow operates in the continuum flow regime and can be treated with the usual fluid expressions. In this regime, ions engage in several collisions during their lifetime with the neutral gas that manifest as either elastic momentum transfer, charge transfer, and other inelastic processes before being lost to the walls or neutralization by recombination[11]. As a result of collisions with neutral gas atoms, it is expected that ions engage in advective transport along with the neutral gas in the axial direction.

We assume that the spatial afterglow retains similar charge phenomena as the plasma. Specifically, the ambipolar electric field is present due to sufficiently high density of charged species, and therefore quasi-neutrality applies up to some critical distance along the direction of the flow. We view positive ions as the centers of the ambipolar potential well due to their sluggish mobility compared to electrons, and electrons stay within the local vicinity of the positive ions, within at least a Debye length, except for electrons in the tail of the energy distribution. The combination of ions being limited to the neutral gas flow field and electrons being limited to the vicinity of ions by action of the ambipolar electric field leads to our assumption that electrons are entrained in the ion advection. We note that this reasoning would not hold when the ambipolar electric field becomes too weak and charged species diffuse freely. The Peclet number, $\mathrm{Pe_D} = v_f L_c / D_a$, was on the order of 10^4 for a flow rate of 1000 sccm through a 3.825 mm ID tube at all pressures (L_c is the characteristic length taken as the distance from the ground electrode to the exhaust port, 68.25 mm), indicating that diffusive transport in the direction of the flow was negligible. We can thus express the flux expression in the axial direction for both electrons and ions as $\Gamma_{i,e} = v_f n_{i,e}$, where v_f is the flow velocity.

Charged species transport occurs in the radial direction and diffusion is the dominant mechanism since radial velocity components were found to be insignificant in CFD analysis. To develop a simplified expression, we reduce the dimensionality by representing radial transport as a lumped loss term. Ions and electrons diffuse from the centerline to the walls with the ambipolar diffusion

coefficient. Thus, the decay model expresses the area-averaged plasma density around the radial center where there is advection in the axial direction. This narrowed scope lends ease of direct comparison to experimental measurements where the length of the Langmuir probe tips is on the order of the flow channel size and the jet.

Finally, we consider three-body recombination as the only charged species loss mechanism in the volume of the spatial afterglow. Three-body recombination occurs between ions and electrons and any third body, including impurities. Here, we assume that the dominant third body is the parent gas. Two-body recombination, involving only ions and electrons, violates conservation of momentum. Provided the assumptions and simplifications explained above are valid, the 1-D steady-state particle balance equation can be expressed as:

$$v_{\mathrm{f}} \frac{dn_{\mathrm{i,e}}}{dz} = -k_{\mathrm{r}} n_{\mathrm{g}} n_{\mathrm{e}} n_{\mathrm{i}} - \frac{2D_{\mathrm{a}}}{\Lambda^2} n_{\mathrm{i,e}} \tag{S21}$$

where $n_{\mathrm{i,e}}$ is the ion and electron density respectively in the plasma bulk, n_{g} is the neutral gas density, v_{f} is the average flow speed, D_{a} is the ambipolar diffusion coefficient, k_{r} is the three-body recombination coefficient, and Λ is the dimension of the flow tube. The ambipolar diffusion coefficient is given by a simplified expression, assuming that the Einstein relation holds and $\mu_{\mathrm{e}} \gg \mu_{\mathrm{i}}$:

$$D_{\mathrm{a}} = \frac{D_{\mathrm{i}}\mu_{\mathrm{e}} + D_{\mathrm{e}}\mu_{\mathrm{i}}}{\mu_{\mathrm{e}} + \mu_{\mathrm{i}}} \approx \frac{\mu_{\mathrm{i}} k_{\mathrm{B}} T_{\mathrm{i}}}{q_{\mathrm{e}}}\left(1 + \frac{T_{\mathrm{e}}}{T_{\mathrm{i}}}\right) = D_{\mathrm{i}}\left(1 + \frac{T_{\mathrm{e}}}{T_{\mathrm{i}}}\right) \tag{S22}$$

where subscripts i and e denote ions and electrons, respectively; q_{e} is the elementary charge, μ is the electrical mobility, D is the intrinsic diffusion coefficient, and T_{i} is the ion temperature, which is assumed to be approximately equal to the gas temperature. The three-body recombination coefficient k_{r} for Ar discharges is expressed by an empirical expression:[12]

$$k_{\mathrm{r}} = 3.86 \times 10^{-44}\left[2 + \frac{11.65}{T_{\mathrm{e}}}\exp\left(\frac{4.11}{T_{\mathrm{e}}}\right)\right] \tag{S23}$$

where k_{r} is in units of $\mathrm{m^6/s}$, and T_{e} is the electron temperature in units of eV. In general, T_{e} and v_{f} may vary along the axial direction. In the temporal afterglow literature, it has been demonstrated that electrons lose energy because of collisions and thus T_{e} decreases as a function of time.[13,14] Applying this reasoning to the electrons in a spatial afterglow, we can expect that T_{e}, and consequently k_{r} and D_{a}, are functions of axial distance. As for v_{f}, gas expansion can occur

depending on the flow geometry and pressure gradients. By assuming local quasi-neutrality, we can avoid solving a system of equations and obtain a single Bernoulli differential equation for the charged species density, n. Assuming no geometrical changes along z, as supported by CFD analysis, we can obtain the general solution for Equation S21:

$$\frac{1}{n} = \exp\left(\frac{2}{\Lambda^2}\int \frac{D_\mathrm{a}}{v_\mathrm{f}} dz\right)\left[C + \int \frac{k_\mathrm{r} n_\mathrm{g}}{v_\mathrm{f}} \exp\left(-\frac{2}{\Lambda^2}\int \frac{D_\mathrm{a}}{v_\mathrm{f}} dz\right) dz\right] \tag{S24}$$

where C is an integration constant. This form is neither compact nor closed and is impractical. A simplification can be made if $D_\mathrm{a}/v_\mathrm{f}$ is approximately constant over the range of interest, yielding:

$$\frac{1}{n} = \exp\left(\frac{2D_\mathrm{a}}{\Lambda^2 v_\mathrm{f}} z\right)\left[C + \int \frac{k_\mathrm{r} n_\mathrm{g}}{v_\mathrm{f}} \exp\left(-\frac{2D_\mathrm{a}}{\Lambda^2 v_\mathrm{f}} z\right) dz\right] \tag{S25}$$

From here, we develop a criterion to determine if $k_\mathrm{r} n_\mathrm{g}/v_\mathrm{f}$ is a much weaker function of z than $\exp(-2D_\mathrm{a}/\Lambda^2 v_\mathrm{f})$ such that a closed form may be obtained from the remaining integral:

$$\left|\frac{d(k_r/v_\mathrm{f})}{dz}\right| \ll \frac{2D_\mathrm{a}}{v_\mathrm{f} n_\mathrm{g} \Lambda^2} \tag{S26}$$

If T_e is approximately constant, the criterion holds, and we can solve for the plasma density, n, along the axial direction of the spatial afterglow with the boundary condition that, at the plasma-afterglow boundary, γ_0, the plasma density is equal to the bulk plasma density, n_p:

$$n(z) = \frac{n_\mathrm{p}}{\left(1 + \frac{k_\mathrm{r} n_\mathrm{g} n_\mathrm{p} \Lambda^2}{2D_\mathrm{a}}\right)\exp\left[\frac{2D_\mathrm{a}}{v_\mathrm{f}\Lambda^2}(z-\gamma_0)\right] - \frac{k_\mathrm{r} n_\mathrm{g} n_\mathrm{p} \Lambda^2}{2D_\mathrm{a}}} \tag{S27}$$

$z = 0$ indicates the location of the upstream tip of the ground electrode, and therefore Equation S27 is valid for $z \geq \gamma_0$, that is, beyond the plasma-spatial afterglow boundary. Equation S27 can be further simplified to give a dimensionless form, η, as a function of dimensionless parameters, λ_c and ϕ:

$$\eta(\xi) = \frac{n}{n_\mathrm{p}} = \frac{1}{\exp(\xi) + \phi^2[\exp(\xi) - 1]} \tag{S28}$$

$$\xi = \frac{z - \gamma_0}{\lambda_\mathrm{c}} = \frac{2D_\mathrm{a}(z - \gamma_0)}{v_\mathrm{f}\Lambda^2} \tag{S29}$$

$$\phi^2 = \frac{k_\mathrm{r} n_\mathrm{g} n_\mathrm{p} \Lambda^2}{2D_\mathrm{a}} = \frac{k_\mathrm{r} n_\mathrm{p} \Lambda^2 p}{2D_\mathrm{a} k_\mathrm{B} T} \tag{S30}$$

The dimensionless form of the solution (Equation S28) expresses the fractional decay as a function of dimensionless distance, ξ, which is the axial distance normalized by a characteristic decay length, λ_c. The dimensionless quantity, ϕ, is analogous to the Thiele modulus in chemical reaction engineering, and here, represents the relative rates of three-body recombination vs. diffusional loss of charge in the spatial afterglow. As expressed in Equation S30, ϕ increases with neutral gas density/pressure if the ideal gas law is applied. The solution (Equation S28) reduces to an exponential decay if $\phi \ll 1$, describing when three-body recombination is insignificant, in agreement with the model constructed by Ferguson et al[15]. This limiting case typically occurs at sufficiently low gas pressures.

Equation S28 can be used to determine a variety of parameters from the slopes of different parts of the decay structure. At low pressure, $\phi \ll 1$, and the slope of the linear $\ln(n/n_p)$ vs. $z-\gamma_0$ gives $\lambda_c = 2D_a/\Lambda^2 v_f$. One can estimate the ambipolar diffusion coefficient if measurements of the plasma density is done at low pressure and if the data appears solely linear on a semi-log plot. This means that electron temperature can also be estimated, albeit more roughly. At high pressure, $\phi \gg 1$, and the slope is the same as the slope above when ξ is sufficiently large. The intercept of this linear portion on the semi-log axis gives ϕ. Therefore, one can estimate the three-body recombination rate coefficient. In summary, this model can be used to estimate parameters of the plasma when the plasma density is measured in the spatial afterglow by some other means (capacitive probe, optical emission spectroscopy combined with collisional radiative models, etc.).

Supplementary Note 6. Electric field in the bulk plasma and spatial afterglow. Similar to the plasma density measurements, we performed measurements of the electric field in both the bulk plasma and the spatial afterglow, the latter being spatially resolved along the axial direction for a few conditions. The calculated values of the reduced electric field were then used as inputs for solving the two-term Boltzmann equation to obtain the electron energy density function (EEDF).

The electric field in the bulk plasma was estimated as a volume-averaged quantity by measuring the discharge voltage across the powered and ground electrode using the RF power probe. Briefly, the power probe measures the root-mean-square voltage, and we take this value to be the discharge voltage when the plasma is switched on, considering that stray components are in parallel to the plasma. Dividing the voltage by the distance between the powered and ground electrode, L_0, yields the electric field, and further dividing by the gas number density, n_g, yields the reduced electric field, *E/N*. We represent *E/N* in units of Townsends (Td) which is equal to 10^{-21} V·m^2. Supplementary Table 1 shows a summary of the measured voltages at different pressures and the calculated values of *E/N*.

The local electric field in the spatial afterglow was measured by rotating the DLP 90° such that the probe tips were aligned vertically (parallel to the flow) as opposed to the horizontal orientation (perpendicular to the flow) used to measure the plasma density. By doing so, the respective probe tips were in different vicinities in the afterglow along the axial direction—the direction of interest in this work—and sensitive to changes in the electric fields, which induce a voltage shift to the DLP traces (see Supplementary Figs. 3 and 5). We determined this voltage shift by locating the point of symmetry in the DLP traces, and then estimated the electric field by dividing the voltage shift by the distance between the probe tips (1.5 mm). We stress that this method does not make any assumption about the energy distribution of electrons, nor the mechanism of ion flux to the probe[5]. Similar to the bulk plasma, the reduced electric field is calculated by dividing the measured electric field by the gas number density. Supplementary Table 2 shows a summary of *E/N* as a function of axial distance in the spatial afterglow at 75 and 300 Torr.

Supplementary Table 1. Summary of experimentally-obtained reduced electric fields in bulk plasma. Voltages measured in bulk plasma as a function of pressure using a RF power probe and corresponding calculated electric fields and reduced electric fields. The gas temperatures, T_g, are also shown which were estimated using a heat transfer model (Equation S31). The distance between the electrodes was 2 cm.

**Pressure / Torr**	**Voltage** / V	**Electric field** / kV m^{-1}	**Temperature** / K	**Reduced electric field** / Td
**10**	390	19.5	300	60.6
**75**	415	20.8	342	9.79
**150**	460	23.0	432	6.86
**300**	415	18.0	655	4.06

Supplementary Table 2. Summary of experimentally-obtained reduced electric fields in the spatial afterglow. Electric fields measured in spatial afterglow as a function of distance using a double Langmuir probe at 75 and 300 Torr, and corresponding calculated reduced electric fields. The gas temperature, T_g, was assumed to be 300 K, and the distance between the probe tips was 1.5 mm. Note that the measured electric field strengths are three orders of magnitude smaller than those calculated for the bulk.

**Pressure / Torr**	**Distance in the afterglow** / mm	**Electric field** / V m^{-1}	**Reduced electric field** / 10^{-3} Td
**75**	3.3	40.2	18.9
	3.9	30.7	14.4
	4.5	8.0	3.8
	4.9	8.0	3.8
**300**	6.5	35.4	8.0
	6.9	26.7	6.0
	7.5	20.5	4.6
	8.1	20.5	4.6
	8.5	1.1	0.24

Supplementary Note 7. Calculation of the electron energy distribution function based on the reduced electric field. Analysis of DLP measurements and the spatial afterglow decay model relied on the assumption that electrons in the afterglow exhibit a Maxwellian energy distribution. To support the existence of a Maxwellian electron energy distribution function (EEDF), we estimated the EEDF by solving the two-term electron Boltzmann equation at 75 Torr and 300 Torr. The measured reduced electric field (Supplementary Table 1), plasma density (Figure 2a in the main text), gas temperature (Supplementary Table 3), and excitation frequency (13.56 MHz) corresponding to the bulk plasma at these pressures, along with the electron–argon collision cross sections (including elastic, electronic excitation, and ionization) from the IST-Lisbon database[16], were used as input data for the code LoKI-B to calculate the EEDF within the bulk plasma[17,18]. The computed EEDFs were compared to Maxwellian and Druyvesteyn distributions at identical values of *E/N* (Supplementary Fig. 8). We focus only on electrons with energies at or below 20 eV, which are relevant to DLP measurements. In general, we found that the EEDFs in this energy range appear to be closer to Druyvesteyn at lower pressures and higher *E/N*, and closer to Maxwellian at higher pressures and lower *E/N*. The EEDF in the spatial afterglow was not calculated because measurements of the plasma density relied on the assumption of a Maxwellian distribution. However, we note that as electrons enter the spatial afterglow, the increasing frequency of electron-neutral collisions (due to a decaying charge density) and the electric field decay (see Supplementary Table 2) suggest that the calculated EEDFs will relax to a Maxwellian, which is consistent with previously reported solutions of the Boltzmann equation for cool electrons under low-field conditions[19]. Additionally, recent studies of temporal afterglows of pulsed argon discharges revealed that deviations from a Maxwellian distribution was inversely proportional to the ionization fraction[20]. Thus, our measurements and calculations support the assumption that the electrons in the spatial afterglow are indeed, or sufficiently close to, Maxwellian.

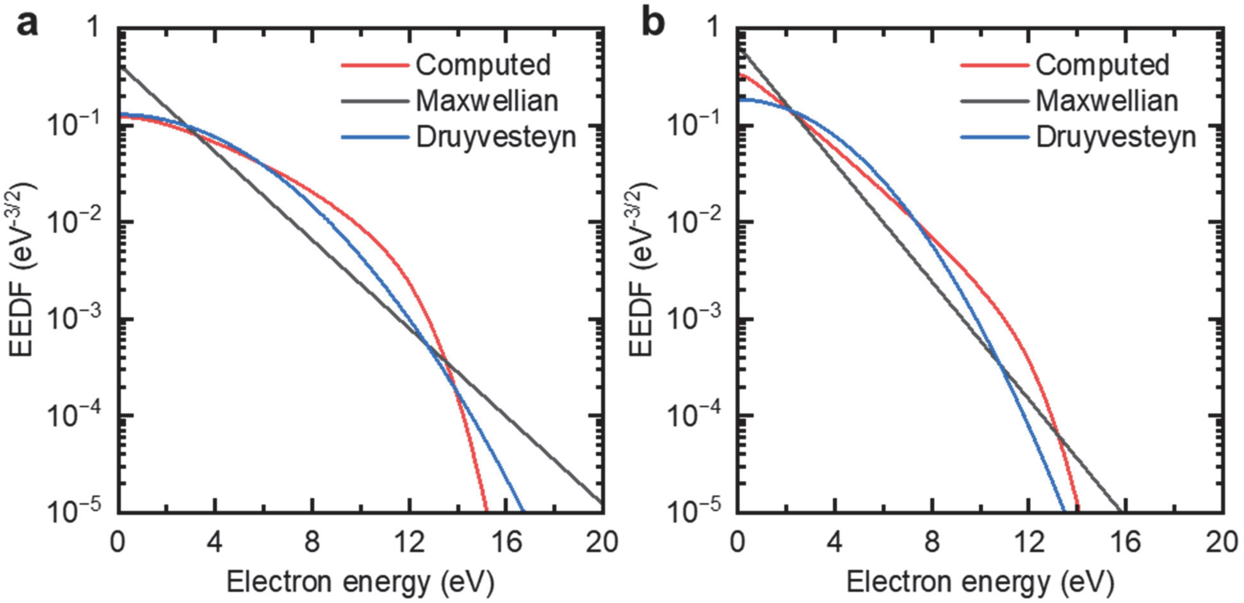

Supplementary Figure 8. Calculated electron energy density functions in the bulk plasma. Electron energy distribution functions at **(a)** 75 Torr and **(b)** 300 Torr computed by solving a two-term electron Boltzmann equation and compared with a Maxwellian and Druvesteyn distribution at the same reduced electric fields. The values of the reduced electric fields used at each pressure are given in Supplementary Table 1.

Supplementary Note 8. Heat transfer model of the bulk plasma. To extend our advection-diffusion-recombination model to high pressures (up to atmospheric), a 1D heat transfer model for the bulk plasma was constructed to estimate the gas temperature at the plasma-afterglow boundary.

We start by considering the spatial dependence of gas temperature in the plasma volume at steady state. We simplify our analysis by assuming that the gas temperature, T_g, is radially uniform and axially inhomogeneous. Potential mechanisms for heat generation include elastic collisions between electrons and neutrals and three-body recombination in the gas volume. Potential mechanisms for heat loss are convective heat transfer to the reactor walls. The 1D heat balance in the bulk plasma can thus be written as:

$$\dot{m}C_\mathrm{p}\frac{dT_\mathrm{g}}{dz} = \pi R_\mathrm{iw}^2\left(\Delta H_\mathrm{rec}k_\mathrm{r}n_\mathrm{g}n_\mathrm{p}^2 + \frac{2m_\mathrm{e}}{M}\varepsilon_\mathrm{mean}\nu_\mathrm{el}n_\mathrm{p}\right) - 2\pi R_\mathrm{iw}h_\mathrm{iw}(T_\mathrm{g} - T_\mathrm{iw}) \quad \text{(S31)}$$

where $\dot{m}$ is the mass flowrate, C_p is the specific heat capacity of the gas, ΔH_{rec} is the energy released from recombination, k_r is the three-body recombination rate coefficient, n_g is the neutral gas density, n_p is the plasma density, m_e is the mass of an electron, M is the mass of a neutral gas atom, ε_{mean} is the average kinetic energy of electrons, ν_{el} is the electron-neutral elastic collision frequency, h_{iw} is the convective heat transfer coefficient on the inner tube walls, R_{iw} is the tube inner radius (equivalent to R and Λ in the power dissipation and spatial afterglow model respectively), and T_{iw} is the temperature of the inner wall. h_{iw} can be calculated from the Nusselt number, Nu, for a laminar flow in a cylindrical tube under constant temperature, assuming that the flow is thermally developed[21]:

$$\mathrm{Nu}_\mathrm{iw} = \frac{2R_\mathrm{iw}h_\mathrm{iw}}{K_\mathrm{Ar}} = 3.66 \quad \text{(S32)}$$

where K_{Ar} is the thermal conductivity of argon. This representation is valid if the inverse of the Graetz numbers are sufficiently large[21]. To confirm this, we artificially varied the constant Nusselt number and found minimal effect on the results.

The inner wall temperature, T_{iw}, is unknown, but it can be determined by assuming steady state and equating the heat fluxes through the reactor walls. We considered the heat fluxes on the inner walls (plasma facing side), within the quartz tube, and on the outer walls (in the ambient). On the inner walls, in addition to convective heat transfer from the gas, surface recombination of charges

must be considered. Here, we adapt the rate of radial transport from the decay model and multiply it by the energy released from recombination to arrive at the heat generated. On the inner walls, the heat flux is:

$$\Gamma_{\mathrm{heat}} = \frac{2\Delta H_{\mathrm{rec}} D_{\mathrm{a}} n_{\mathrm{p}}}{R_{\mathrm{iw}}} + h_{\mathrm{iw}}\left(T_{\mathrm{g}} - T_{\mathrm{iw}}\right) \tag{S33}$$

where D_{a} is the ambipolar diffusion coefficient. Thermal conduction occurs through the solid reactor walls and depends on the difference between the inner and outer wall temperatures. However, we can evaluate the dimensionless Biot number to determine whether two separate wall temperatures are necessary. The Biot number compares the thermal resistance from convection in the gas phase to conduction in the solid phase. We found that the Biot number is significantly less than one up to 3000 K, allowing us to treat the problem with a singular wall temperature, T_{iw}.

On the outside of the cylindrical reactor, heat is dissipated through natural convection and thermal radiation. The heat flux on the outer wall is:

$$\Gamma_{\mathrm{heat}} = \sigma_{\mathrm{Q}}(T_{\mathrm{iw}}^4 - T_{\infty}^4) + h_{\mathrm{ow}}(T_{\mathrm{iw}} - T_{\infty}) \tag{S34}$$

where σ_{Q} is the Stefan-Boltzmann constant multiplied by the emissivity of quartz (0.93), h_{ow} is the free convection heat transfer coefficient, and T_{∞} is the ambient temperature (300 K). Similar to h_{iw}, h_{ow} can be calculated from the Nusselt number, but the Nusselt number varies with the Rayleigh number, Ra, which is the ratio between thermal transport via diffusion to thermal transport via convection:

$$\mathrm{Nu}_{\mathrm{ow}} = \frac{2R_{\mathrm{ow}} h_{\mathrm{ow}}}{K_{\mathrm{air}}} = C(\mathrm{Ra})^n \tag{S35}$$

where C and n are empirical constants that depend on the value of Rayleigh number. The left-hand side of Equation S35 describes the Nusselt number for a vertical cylinder[21]. The Rayleigh number is calculated by the expression:

$$\mathrm{Ra} = \frac{16 g R_{\mathrm{ow}}^3}{\nu\alpha} \frac{T_{\mathrm{iw}} - T_{\infty}}{T_{\mathrm{iw}} + T_{\infty}} \tag{S36}$$

where g is the acceleration due to gravity, ν is the kinematic viscosity of air, and α is the thermal diffusivity of air.

Setting the heat flux on the inside of the tube (Equation S33) equal to the heat flux on the outside of the tube (Equation S34), the wall temperature, T_{iw}, can be determined as a function of the gas temperature, T_g, and the heat balance (Equation S31) can be solved as a simple initial value problem. Results are shown in Supplementary Table 3 and Supplementary Figures 9-11, where 'Forced convection' occurs in the tube; 'Surface recombination' occurs on the inner walls of the tube; 'Solid conduction' occurs across the tube walls; 'Thermal radiation' and 'Free convection' occurs from the outer walls to the ambient.

Supplementary Table 3. Summary of calculated gas temperatures at the plasma-afterglow boundary. Gas temperatures calculated by a heat transfer model at the plasma-afterglow boundary for various pressures, gas flow rates, and tube (inner) diameters.

**Pressure / Torr**	**Flowrate** / sccm	**Inner diameter** / mm	**Temperature** / K
**75**	1000	3.85	340
**300**	1000	3.85	680
**760**	1000	3.85	1470
**760**	500	3.85	2060
**760**	2500	3.85	960
**760**	1000	1.18	570
**760**	1000	7.02	2540

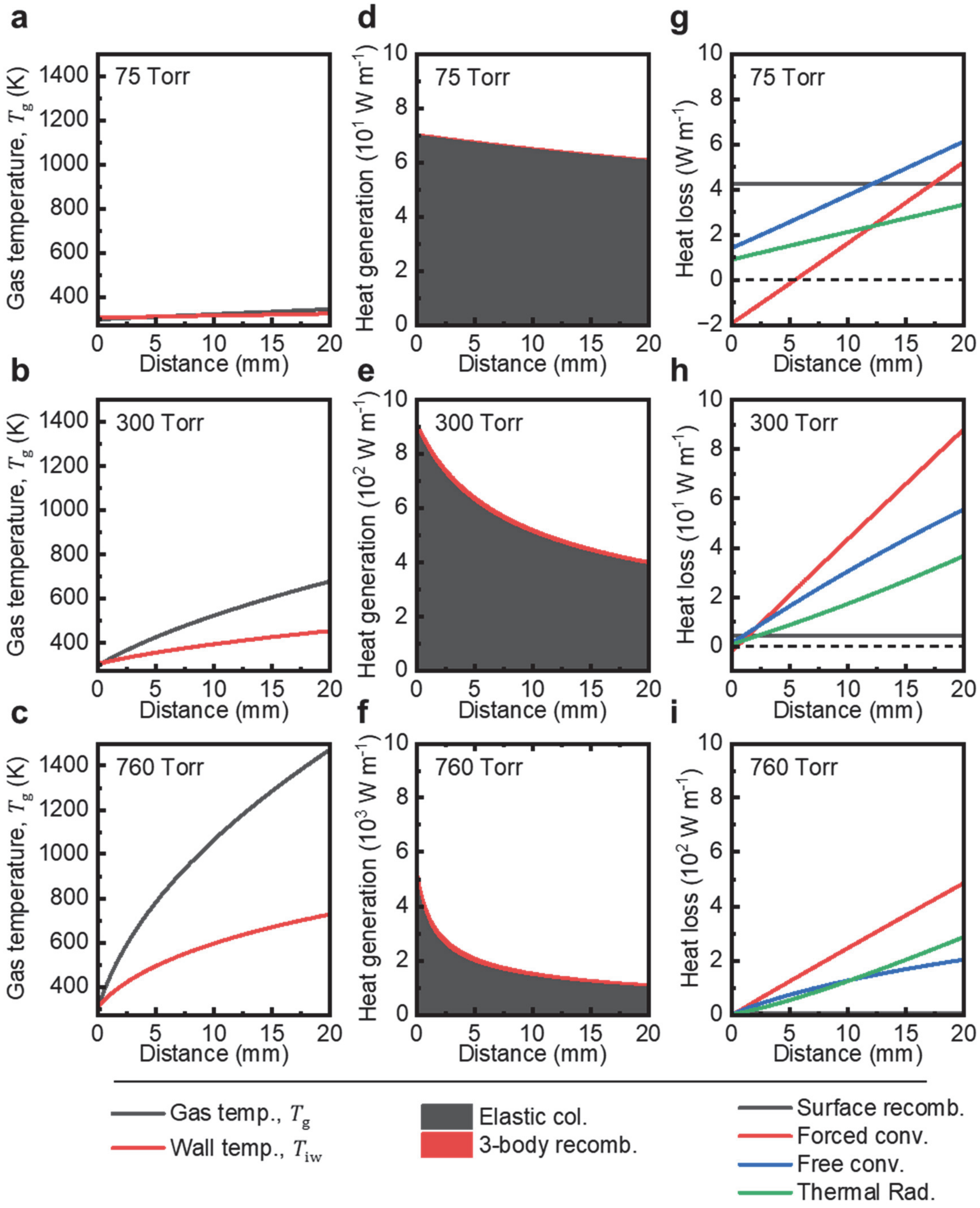

Supplementary Figure 9. Heat transfer model results for bulk plasma as a function of pressure. (a-c) Gas, inner wall, and outer wall temperatures as a function of distances at 75, 300, and 760 Torr, respectively. **(d-f)** Volumetric heat generation as a function of distance by elastic collisions and three-body recombination at 75, 300, and 760 Torr, respectively. **(g-i)** Heat loss per unit length to the wall as a function of distance by various mechanisms at 75, 300, and 760 Torr, respectively. Negative values indicate heat flow into the gas. The flow rate was 1000 sccm and the inner tube diameter was 3.85 mm.

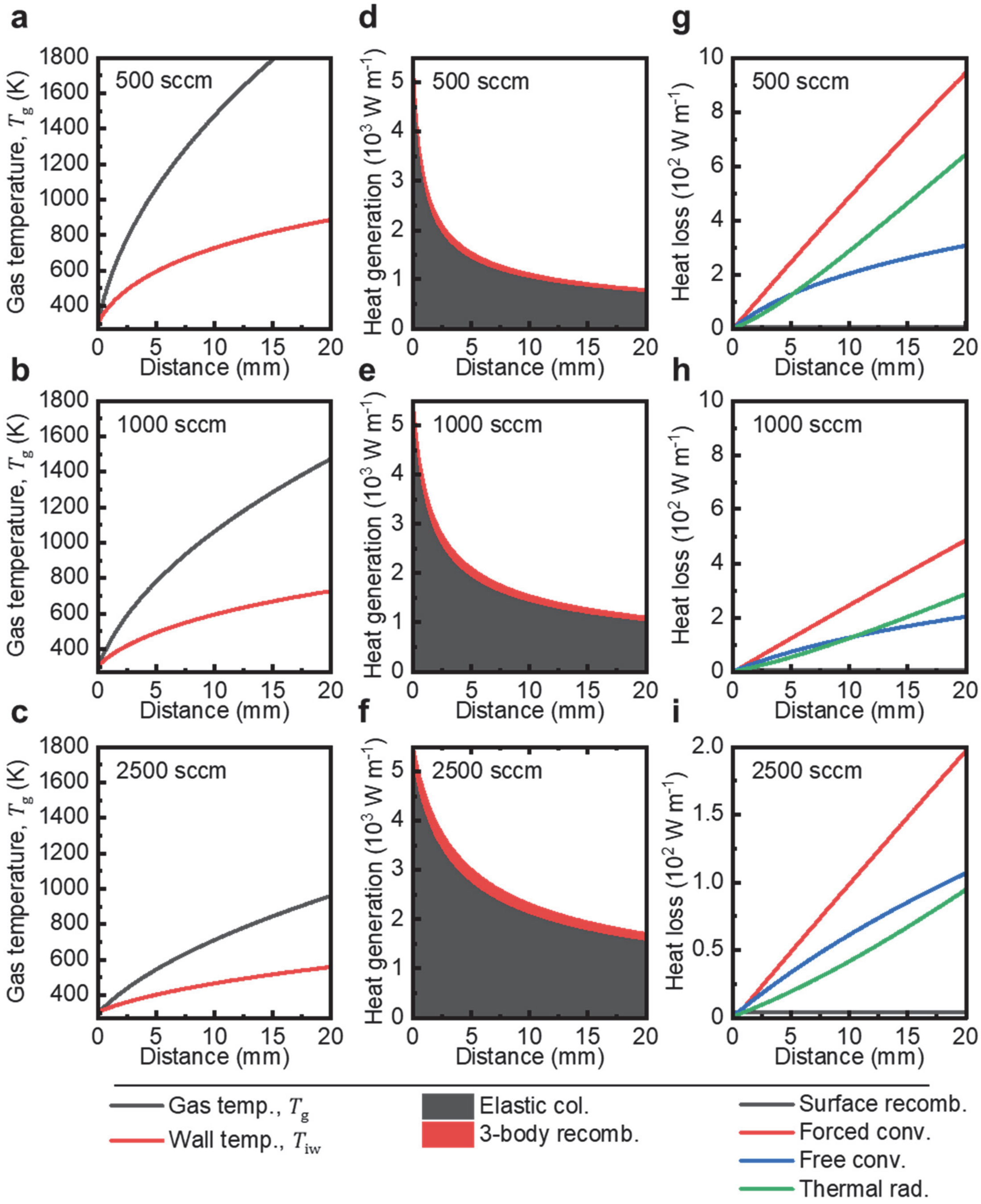

Supplementary Figure 10. Heat transfer model results for bulk plasma as a function of flow rate. (a-c) Gas, inner wall, and outer wall temperatures as a function of distance at 500, 1000, and 2500 sccm, respectively. **(d-f)** Volumetric heat generation as a function of distance by elastic collisions and three-body recombination at 500, 1000, and 2500 sccm, respectively. **(g-i)** Heat loss per unit length to the wall as a function of distance for various mechanisms at 500, 1000, and 2500 sccm, respectively. The pressure was 760 Torr and the inner tube diameter was 3.85 mm.

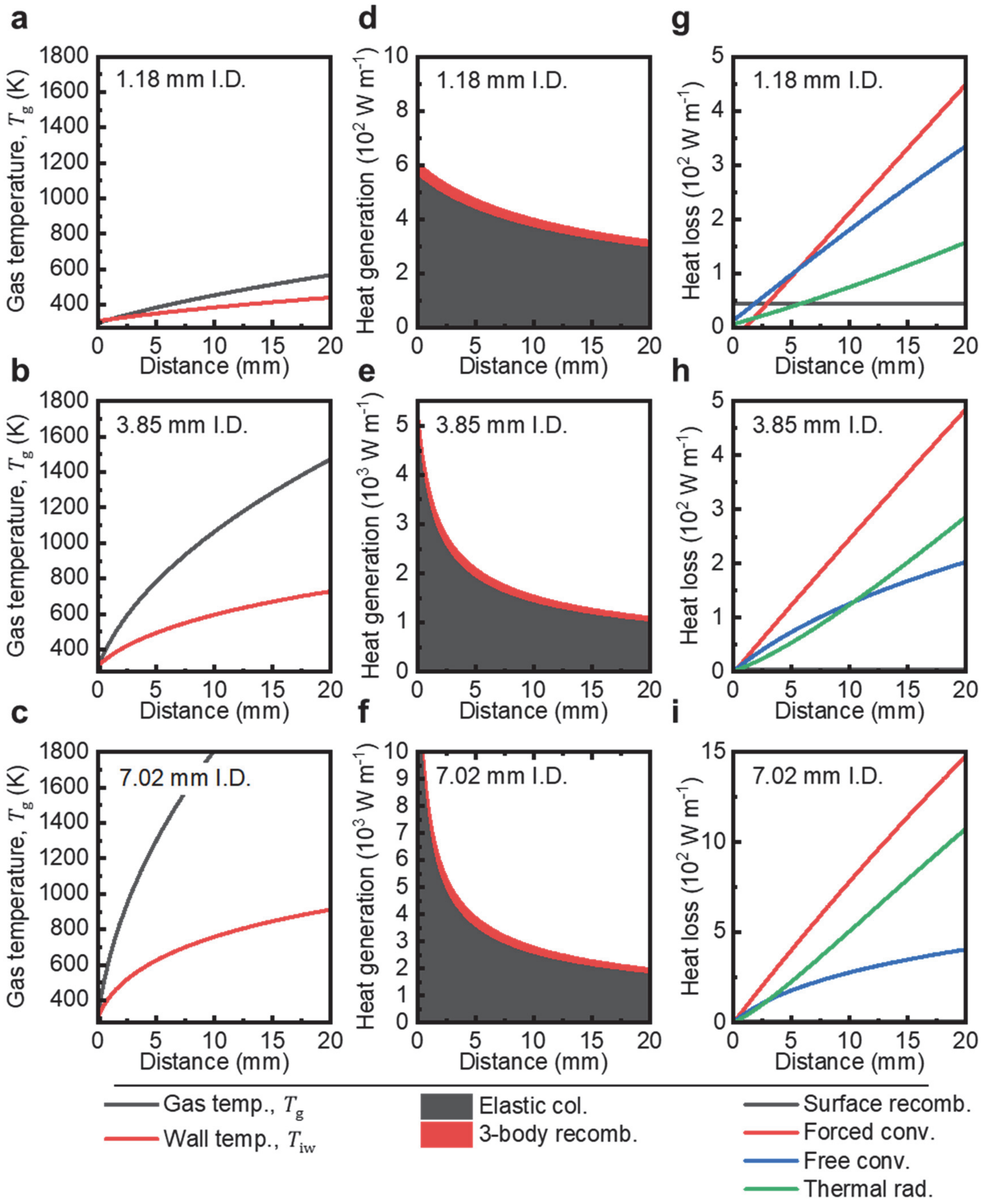

Supplementary Figure 11. Heat transfer model results for bulk plasma as a function of tube inner diameter. (a-c) Gas, inner wall, and outer wall temperatures as a function of distance at 1.18, 3.85, and 7.00 mm inner tube diameter, respectively. **(d-f)** Volumetric heat generation as a function of distance by elastic collisions and three-body recombination at 1.18, 3.85, and 7.00 inner tube diameter, respectively. **(g-i)** Heat loss per unit length to the wall as a function of distance for various mechanisms at 1.18, 3.85, and 7.00 mm inner tube diameter, respectively. The pressure was 760 Torr and 1000 sccm.

Supplementary Note 9. Calculation of ξ_{crit} at diffusion and recombination limits. The critical distance, ξ_{crit}, can be derived for limiting cases where either diffusion or recombination is the dominant loss mechanism of charged species in the afterglow. To calculate ξ_{crit} in the diffusion limit, the governing transport equation is solved by only considering diffusive losses,

$$v_{\mathrm{f}} \frac{dn}{dz} = -\frac{2D_{\mathrm{a}}}{\Lambda^2} n \tag{S37}$$

and with the same boundary condition used in the original model, producing a solution in the form of an exponential decay:

$$\eta(\xi) = \frac{n(\xi)}{n_{\mathrm{p}}} = \exp\left[-\frac{2D_{\mathrm{a}}}{v_{\mathrm{f}}\Lambda^2}(z - \gamma_0)\right] = \exp(-\xi) \tag{S38}$$

In the diffusion limit, the Debye length, λ_D, as a function of axial distance can be represented as:

$$\lambda_{\mathrm{D}}^{\mathrm{diff}}(\xi) = \lambda_{\mathrm{Dp}} \exp\left({}^{\xi}/_{2}\right) \tag{S39}$$

where $\lambda_{Dp} = (\epsilon_0 T_e / q_e n_p)^{1/2}$ is the Debye length in the bulk plasma, q_e is the elementary charge, and ϵ_0 is the vacuum permittivity. Following the derivation presented in the main text, the critical distance, ξ_{crit}, in the diffusion dominant case is:

$$\xi_{\mathrm{crit}}^{\mathrm{diff}} = 2 \ln\left({}^{\chi_{\mathrm{p}}}/_{\chi_{\mathrm{crit}}}\right) \tag{S40}$$

where χ_p corresponds to bulk plasma conditions and χ_{crit} is the critical value of 30. This expression can also be derived by setting ϕ = 0 in Equation 4 in the main text. To calculate ξ_{crit} in the recombination limit, we can modify Equation 4 by presuming the Thiele modulus, ϕ, is much greater than unity. By doing so and simplifying the expression, we obtain:

$$\xi_{\mathrm{crit}}^{\mathrm{rec}} = \ln\left[\left({}^{\chi_{\mathrm{p}}}/_{\chi_{\mathrm{crit}}\phi}\right)^2 + 1\right] \tag{S41}$$

Supplementary References

1. Johnson, E. O. & Malter, L. A Floating Double Probe Method for Measurements in Gas Discharges. *Physical Review* **80**, 58 (1950).
2. Brockhaus, A., Borchardt, C. & Engemann, J. Langmuir probe measurements in commercial plasma plants. *Plasma Sources Science & Technology* **3**, 539 (1994).
3. Castro, R. M., Cirino, G. A., Verdonck, P., Maciel, H. S., Massi, M., Pisani, M. B. & Mansano, R. D.. A Comparative Study of Single and Double Langmuir Probe Techniques for RF Plasma Characterization. *Contributions to Plasma Physics* **39**, 235–246 (1999).
4. Peterson, E. W. & Talbot, L. Collisionless electrostatic single-probe and double-probe measurements. *American Institute of Aeronautics and Astronautics Journal* **8**, 2215–2219 (2012).
5. Cozens, J. R. & Von Engel, A. Theory of the Double Probe at High Gas Pressure. *International Journal of Electronics* **19**, 61-68 (1965).
6. Wild, J., Kudrna, P., Tichy, M., Nevrly, V., Strizik, M., Bitala, P., Filipi, B. & Zelinger, Z. Electron Temperature Measurement in a Premixed Flat Flame Using the Double Probe Method. *Contributions to Plasma Physics* **52**, 692-698 (2012).
7. Liu, C. J., Wang, J. X., Yu, K. L., Eliasson, B., Xia, Q., Xue, B. Z. & Zhang Y. H. Floating double probe characteristics of non-thermal plasmas in the presence of zeolite. *Journal of Electrostatics* **54**, 149-158 (2002).
8. Iza, F. & Lee, J. K. Particle-in-cell simulations of planar and cylindrical Langmuir probes: Floating potential and ion saturation current. *Journal of Vacuum Science & Technology A: Vacuum, Surfaces, and Films* **24**, 1366 (2006).
9. Mason, E. A. & McDaniel, E. W. *Transport Properties of Ions in Gases*. (Wiley, 1988).
10. Raizer, Y. P. *Gas Discharge Physics*, *First Edition*. (Springer-Verlag, 1991).
11. Lieberman, M. A. & Lichtenberg, A. J. *Principles of Plasma Discharges and Materials Processing: Second Edition*. (Wiley, 2005).
12. Owano, T. G., Kruger, C. H. & Beddini, R. A. Electron-ion three body recombination coefficient of argon. *AIAA 22nd Fluid Dynamics, Plasma Dynamics and Lasers Conference, 1991* (1991).
13. Couëdel, L. Temporal dusty plasma afterglow: A review. *Frontiers in Physics* **10**, 913 (2022).

14. Couëdel, L., Samarian, A. A., Mikikian, M. & Boufendi, L. Dust charge distribution in complex plasma afterglow. *Europhysics Letters* **84**, 35002 (2008).

15. Ferguson, E. E., Fehsenfeld, F. C. & Schmeltekopf, A. L. Flowing Afterglow Measurements of Ion-Neutral Reactions. *Advances in Atomic, Molecular and Optical Physics* **5**, 1–56 (1969).

16. Alves, L. L. The IST-LISBON database on LXCat. in *Journal of Physics: Conference Series* **565**, 012007 (2014).

17. Tejero-Del-Caz, A. Guerra, V., Goncalves, D., Lino da Silva, M., Marques, L., Pinhao, N., Pintassilgo, C. D. & Alves, L. L. The LisbOn KInetics Boltzmann solver. *Plasma Sources Science & Technology* **28**, 043001 (2019).

18. Tejero-Del-Caz, A., Guerra, V., Pinhao, N., Pintassilgo, C. D. & Alves, L. L. On the quasi-stationary approach to solve the electron Boltzmann equation in pulsed plasmas. *Plasma Sources Science & Technology* **30**, 065008 (2021).

19. Cherrington, B. E. *Gaseous Electronics and Gas Lasers*, vol. 94, (Pergamon, 1979).

20. Carbone, E., Sadeghi, N., Vos, E., Hubner, S., van Veldhuizen, E., van Dijk, J., Nijdam, S. & Kroesen, G.. Spatio-temporal dynamics of a pulsed microwave argon plasma: Ignition and afterglow. *Plasma Sources Science & Technology* **24**, 015015 (2015).

21. Incropera, F. P., DeWitt, D. P., Bergman, T. L. & Lavine, A. S. *Principles of Heat and Mass Transfer*, vol. 6, (Wiley, 2013).
